# Ethical Issues in Social Science Research Employing Big Data

**DOI:** 10.1007/s11948-022-00380-7

**Published:** 2022-06-15

**Authors:** Mohammad Hosseini, Michał Wieczorek, Bert Gordijn

**Affiliations:** 1 Feinberg School of Medicine, Northwestern University, Chicago, USA; 2 Institute of Ethics, Dublin City University, Dublin, Ireland

**Keywords:** Research Ethics, Research Integrity; Big Data, Social Science, Computational Social Science, Open Science

## Abstract

This paper analyzes the ethics of social science research (SSR) employing big data. We begin by highlighting the research gap found on the intersection between big data ethics, SSR and research ethics. We then discuss three aspects of big data SSR which make it warrant special attention from a research ethics angle: (1) the interpretative character of both SSR and big data, (2) complexities of anticipating and managing risks in publication and reuse of big data SSR, and (3) the paucity of regulatory oversight and ethical recommendations on protecting individual subjects as well as societies when conducting big data SSR. Against this backdrop, we propose using David Resnik’s research ethics framework to analyze some of the most pressing ethical issues of big data SSR. Focusing on the principles of honesty, carefulness, openness, efficiency, respect for subjects, and social responsibility, we discuss three clusters of ethical issues: those related to methodological biases and personal prejudices, those connected to risks arising from data availability and reuse, and those leading to individual and social harms. Finally, we advance considerations to observe in developing future ethical guidelines about big data SSR.

## Introduction

This paper explores ethical issues of employing big data^1^ in social science research (SSR) with a specific focus on how these practices challenge the integrity and ethics of research. In recent years, the research community has witnessed the introduction of new technologies that collect and process big data. Social scientists have particularly benefited from these developments as their research increasingly generates big data sets or reuses existing ones such as those collected by public institutions and federal agencies (Foster et al., 2016, pp. 1–9), those generated and collected by social media platforms (Townsend & Wallace, 2016), e.g., Facebook analytics, and those generated by developers of digital devices and services (Lazer et al., 2009), e.g., Google Trends.

With the increasing use and reuse of big data sets in SSR, new ethical concerns emerge that need to be recognized, communicated to the research community, and mentioned in research ethics guidelines and protocols. Exploring these issues becomes more relevant when we consider the surge of studies that source their data from countries with dissimilar standards or employ publicly available data (e.g., harvested from social media platforms) without addressing ethical issues (OECD, 2016). As shown in a recent paper, 64% of studies (n = 132) that used big data “did not discuss ethical issues, mostly claiming the data were publicly available” (Stommel & de Rijke, 2021, p. 1).

Despite the significance of the topic from a research ethics and integrity perspective, an exploratory scoping search conducted for this study showed that the published literature has paid little attention to the challenges posed by big data SSR for upholding the norms of research ethics and integrity (for this purpose, the Web of Science core collection was searched on 18/06/2021 with the following string “social science*” AND “big data” AND “ethics”. Using this string yielded 22 items, only one of which exclusively discussed ethics of big data SSR). In fact, a recent review of the literature (n = 892) concludes that big data ethics are mainly discussed in relation to health and technology (Kuc-Czarnecka & Olczyk, 2020). This could be due to the historical roots of the discipline of ethics and its closer ties with biomedical sciences (Resnik, 2015), or big data’s closer ties to discussions about technology as the “term refers to the vast amounts of digital data which are being produced in technologically and algorithmically mediated practices” (Richterich, 2018, p. 4).

In contexts where big data SSR is discussed, authors have raised concerns about consent, privacy, potential harm to research subjects and data ownership (Lipworth et al., 2017; Lunshof et al., 2008; Mittelstadt & Floridi, 2016; Metcalf & Crawford, 2016; Rothstein, 2015; Starkbaum & Felt, 2019; Zimmer, 2018). Sometimes the methodological problems associated with the move to a data-driven/computational SSR paradigm have received more attention than ethical aspects (with some notable exceptions such as Weinhardt’s study (2020) and Salganik’s book (2017), but even within these contributions, the ethical issues are either not analyzed systematically or the impact of the interpretative nature of SSR on ethical issues is neglected^2^). Some existing studies develop tools for analyzing big data in SSR or note difficulties that arise when big data analysis methods developed for biomedical/engineering purposes are employed in SSR. Authors of these studies mostly mention, but not elaborate on, challenges related to privacy and consent (Chang et al., 2014; Connelly et al., 2016; González-Bailón, 2013; Liu, 2016) or legal and liability issues (Bender et al., 2016).

Furthermore, although papers in two special issues of the *American Behavioral Scientist* (Volume 63, Issue 5 and 6, 2019) and a special issue of *Social Science Computer Review* (Volume 38, Issue 1, 2020) provide useful perspectives on the ethical issues of SSR^3^, only one of these contributions uses a normative framework to provide a systematic analysis of ethical issues. These papers discuss big data’s impact on social interpretations and context (Camfield, 2019; Feldman & Shaw, 2019; Frey et al., 2020; Hesse et al., 2019), data representativeness (Hargittai, 2020), data accuracy and inclusiveness (Popham et al., 2020), data sharing and replicability (Mannheimer et al., 2019; Sterett, 2019), press and personal freedom (Shahin & Zheng, 2020) as well as issues related to the prioritization of big data as a source and the impact of big data tools on research questions and results (Hesse et al., 2019; Mauthner 2019). Hossain & Scott-Villiers (2019) explicitly base their analysis on an ethical framework, but since their adopted approach only captures qualitative SSR (similar to other papers in the *American Behavioral Scientist* special issues), they problematize relationships between researchers and subjects based on the quality of relationships without discussing biases/prejudices. Thus, we believe that applying a research ethics framework and paying specific attention to the interpretive nature of SSR in this paper, expands the scope of the current debate about big data SSR.

In what follows we first distinguish three reasons why ethics of big data SSR matters. Then we employ David Resnik’s research ethics framework to systematically analyze the ethics of big data SSR. Consequently, we advance suggestions for researchers, data repositories and research institutions to minimize the likelihood of ethical issues in big data SSR.

## Three Reasons for Ethical Concerns About Big Data SSR

Without claiming to be exhaustive, we highlight three factors that motivated our concerns about the ethics of big data SSR: (1) the interpretative aspects of SSR provide fertile grounds for different forms of bias, (2) anticipating and managing risks in publication and reuse of big data SSR is complicated, and (3) the paucity of regulatory oversight and ethical recommendations on protecting subjects and societies when conducting big data SSR.

1) While some approaches to social science define it as a discipline concerned with studying *facts* about society to formulate theories and predictions about it (Popper, 1961), we endorse the view that social sciences interpret societies’ norms and practices through the lens of values and beliefs held by researchers (Richardson & Fowers, 1998; Taylor, 1971). Especially in cases where SSR focuses on subjective concepts and phenomena such as culture, behavior, social relations, shared imagination and beliefs, results are markedly interpretative and reflect the cultural context, the historical circumstances in which they are produced, as well as the worldviews of involved researchers (Feldman & Shaw, 2019; Taylor, 1971). Although interpretative practices allow us to make sense of the social world, they can expose research and its outcomes to external factors such as researchers’ moral beliefs, prejudices, stereotypes, values or even the used language. Using big data in SSR further complicates this problem because big data technologies can potentially affix problematic interpretations into research when third-party technology and services are employed in data collection or analysis (Barocas & Selbst, 2016). Of course, sometimes this problem is exacerbated by using big data processing techniques designed for STEM disciplines (arguably a misfit for studying people, beliefs and behavior).^4^ Moreover, a positivist view of data (i.e., data as an objective entity), can be in conflict with the interpretative aspects of SSR (Hesse et al., 2019).

2) There is no such thing as *raw* data or big data sets that simply represent facts (Gitelman, 2013; Barrowman, 2018). Arguably, big data is always already interpreted by those who generated data sets or, in the case of automatically created data sets, by employed algorithms and their designers. Researchers engaged with pre-processed data or data reuse could further divorce it from rawness by attributing meaning to it over the course of subsequent analyses. These future uses and analyses are not always in line with data generators’ objectives. Therefore, dissemination of big data SSR results may involve risks that are hard to identify/manage even for researchers strongly determined to uphold research ethics and integrity norms. Furthermore, algorithmic tools that analyze and interpret big data SSR might influence results by operating under assumptions that are not endorsed by researchers or their subjects (e.g., what should be considered normal in each population, cf. Neff & Nafus, 2016, 48–49). Indeed, big data sets could reveal unforeseen connections, patterns and information, making it difficult for investigators to anticipate the outcomes and consequences of future analyses (Mittelstadt & Floridi, 2016). These challenges not only threaten methodological soundness, but also have ethical implications when big data SSR generates unpredictable results that could justify discrimination, symbolic violence^5^ and other harmful practices that are difficult to anticipate when research is being designed, conducted or published. In particular, since data literacy is a specialized skill unequally possessed by researchers, policymakers, and the public (Wolff et al., 2016), results produced by big data SSR might confuse various stakeholders (Pangrazio & Sefton-Green, 2020) about their intended purpose or their actual meaning (boyd & Metcalf, 2014).

3) Methods and devices employed to collect health-related information are subjected to strict regulatory oversight and their reliability is demonstrated in elaborate trials (Kramer et al., 2020). Such stringent requirements are not applied to SSR, and if applied, they are considered a misfit (National Research Council, 2003). Using a biomedical understanding of ethical principles and issues “such as avoiding harm and doing good, informed consent, confidentiality, etc.” for SSR, could result in misjudging the impact of SSR on research subjects and societies (Gurzawska & Benčin, 2015, p. 5). Accordingly, big data SSR could serve as a justification for discriminatory policy decisions against research subjects or create and reinforce harmful stereotypes about social groups. Especially since many researchers engaged in big data SSR are not social scientists by training, they might be insufficiently trained/prepared to anticipate likely harms arising from SSR (Hesse et al., 2019). Experts have argued that one reason why these issues are not adequately addressed during the design, data collection, analysis and publication of big data SSR is that available ethical frameworks are not well-equipped to address them (Boyd, 2017).^6^ In addition, regulatory bodies, Institutional Review Boards (IRBs) and Research Ethics Committees, are inadequately equipped to evaluate ethical issues of big data SSR (Favaretto et al., 2020; Vitak et al., 2017). It is challenging to capture ethical issues of big data SSR as they evolve alongside big data technologies. The necessity to *continuously revise* guidelines, even those that are developed for a specific data collection method e.g., Internet Research: Ethical Guidelines (franzke et al., 2020) demonstrates the dynamic landscape of this domain and calls for the improvement of current guidelines (Hollingshead et al., 2021).

## Big Data SSR Through the Lens of Resnik’s Principles

To explore the ethical issues of big data SSR in a systematic manner, we employ the normative framework developed in David Resnik’s *Ethics of science* (2005). This framework consists of twelve principles: honesty, carefulness, openness, freedom, credit, education, social responsibility, legality, opportunity, mutual respect, efficiency, and respect for subjects. Although all twelve principles are relevant to big data SSR, in our analysis we focus on the six principles of honesty, carefulness, openness, efficiency, responsibility and respect for subjects. Employing the six mentioned principles in three pairs enables us to systematically explore what we deem to be the three most pressing reasons for ethical concern in the context of big data SSR. In what follows we discuss three clusters, each addressing two principles. These include ethical issues about bias (the principles of honesty and carefulness), risks relating to publication and reuse of big data (the principles of openness and efficiency) and ethical concerns about individuals and societies (the principles of social responsibility and respect for subjects).

First, Resnik’s framework allows us to make a distinction between two types of bias. One type (discouraged by the principle of carefulness) pertains to biases that might be embedded in methodologies and techniques used in research processes (what we call methodological biases, which as explained in the previous section are pronounced in using big data). The second type (discouraged by the principle of honesty) is related to researchers’ personal values, worldviews, preferences, used language, etc., that may affect their observations, inferences or conclusions (what we call prejudice). Given the aforementioned weaknesses (e.g., misfit) of big data analysis methods for SSR, and the hermeneutic nature of SSR, making a distinction between these two types of bias helps articulating ethical issues more specifically. These two forms of bias are discouraged by the principles of honesty and carefulness and are explored in detail in Sect. 3.1:

Honesty: “scientists should not fabricate, falsify, or misrepresent data or results. They should be objective, *unbiased*, and truthful in all aspects of the research process” [emphasis added] (Resnik, 2005, p. 48).

Carefulness: “Scientists should avoid errors in research, especially in presenting results. They should minimize experimental, methodological, and human errors and avoid self-deception, *bias*, and conflicts of interest” [emphasis added] (Resnik, 2005, p. 51).

Second, Resnik’s principles of openness and efficiency are also particularly useful in exploring ethical issues related to the publication/reuse of big data and the associated risks.

Openness: “Scientists should share data, results, methods, ideas, techniques, and tools. They should allow other scientists to review their work and be open to criticism and new ideas” (Resnik, 2005, p. 52).

Efficiency: “Scientists should use resources efficiently” (Resnik 2005, p. 60).

When it comes to using big data, the principles of openness and efficiency are not only connected but also inseparable, making both relevant to exploring the risks of big data publication and reuse. While openness of data enables efficient use of resources (e.g., data reuse), efficient use of resources requires openness of data. However, as Sect. 3.2 shall demonstrate, attempts to uphold both in the context of big data SSR contributes to specific risks.

Third, Resnik’s framework is developed with the recognition of social impacts of SSR (e.g., influence of results on social and political agendas) in addition to personal harms (Resnik, 2005, p. 133). Accordingly, it allows us to identify and explore two forms of ethical concerns, one related to research subjects (e.g., dignity) and one to societies (e.g., harms to society), both formulated as normative principles:

Respect for subjects: “scientists should not violate rights or dignity when using human subjects in experiments” (Resnik, 2005, p. 61).

Social responsibility: “scientists should avoid causing harms to society and they should attempt to produce social benefits. Scientists should be responsible for the consequences of their research and they should inform the public about those consequences” (Resnik, 2005, p. 57).

As will be shown in Sect. 3.3, in the context of big data SSR, respect for subjects might not necessarily prevent harms to societies and attempts to uphold both of these principles might not always succeed.

### Prejudices and Biases

Recent developments in big-data-generating technologies have opened new possibilities for social scientists, some of which might infuse new forms of prejudice and bias into research outcomes. Prejudices and biases discussed in this section not only hinder researchers’ adherence to the principles of honesty and carefulness but might be so subtle that even the most diligent researchers might be unable to neutralize them.

While researchers have more control over methods used to generate original data sets (compared with reusing existing data sets), they cannot always identify biases introduced by technologies they employ. Although this difficulty is present in all kinds of research to a degree, we argue that the sheer variety, velocity and volume of information in big data sets make researchers’ dependence on technology greater while reducing their control over technologies’ impact, thus, exacerbating ethical issues. Accordingly, by employing data sets that were generated with the help of technology/services/software delivered by third parties (whether generating their own datasets or reusing available datasets), social scientists might face specific ethical challenges regarding bias. Depending on the stage(s) wherein third-party technology is used, their inherent biases might corrupt data collection, study designs and analysis with, for example, lack of considerations for relevant characteristics of respondents (e.g., membership of vulnerable groups or endorsement of certain political views). These challenges might hamper social scientists’ ability to identify, let alone avoid methodological biases as demanded by the principle of carefulness. To articulate some of these biases more clearly, we will use self-tracking^7^ and crowdsourcing platforms employed in SSR as examples that complicate researchers’ adherence to principles of honesty and carefulness.

I) In some SSR contexts (e.g., psychology, anthropology, sport and health sociology), researchers employ automated data collection devices (e.g., self-tracking devices) worn/used by research subjects to explore movement, health and/or productivity (Neff & Nafus, 2016; Lupton, 2016). These data collections are not always accurate; hence, resulted conclusions might not be as objective and unbiased as they appear. Research shows that self-tracking devices cannot always reliably detect particular kinds of movement, which leads them to inflate/underestimate activity metrics, while still framing them as accurate and objective (Hoy, 2016; Piwek et al., 2016; Moore & Piwek, 2017). Moreover, even if self-tracking devices could (accurately) capture all possible movements, their designers might categorize and understand these in ways different than researchers. For example, since the definition of an intense workout and the recommended activity levels for each individual remain rather ambiguous, different technologies use dissimilar parameters to define specific variables. Consequently, devices from two different manufacturers might provide altogether different results for the same subject, even in measurements as seemingly uncomplicated as step-counting (Crawford et al., 2015). According to Crawford and colleagues, this issue becomes even more pronounced when complex parameters, such as the differences between light and deep sleep are considered. These parameters might be important information for social scientists investigating for example, the relationship between physical and mental health and the quality of the neighborhood wherein research subjects live (Hale et al., 2013). Although the objectivity and accuracy of such results cannot always be fully trusted, upon publication (and partly due to varied levels of data literacy of different stakeholders, as mentioned in Sect. 2), results can be interpreted (and reproduced in popular media) with blind faith because they are expressed numerically, and therefore, resemble objective measurement (Mills, 2018).

Furthermore, it is possible that collected data lacks contextual information because researchers might be unable/unmotivated to examine and disclose contextually relevant information that impacted data sets. For example, even though self-tracking data about geolocation and physical activity might be highly beneficial for a study that investigates people’s mobility and public health risks, such data might not necessarily provide all the contextual information required to make accurate conclusions about the studied cohort. A one-size-fits-all approach of data-collection devices does not account for variables such as childcare responsibilities or injury history of research subjects, which can influence the extent and intensity of daily movements (Neff & Nafus, 2016; Selke, 2016).^8^ Consequently, while some researchers might be inclined to make seemingly objective and science-based conclusions when employing big data in SSR, a careful evaluation of what information is missing from the used data sets and the implications of missing such information for the overall conclusions could reveal undisclosed limitations and biases (cf. Camfield, 2019).

II) Algorithmic bias and limitations of third-party technologies remain mostly undisclosed; hence, researchers cannot always employ measures to offset biases. Data-generating devices process collected information using algorithms that operate in line with instructions and assumptions of their developers. As designers of algorithmic tools might be unaware of their own presuppositions and prejudices or they might not actively take steps to avoid biases in designing algorithms, many contemporary technologies have been demonstrated to exhibit various forms of algorithmic bias (Friedman & Nissenbaum, 1996; Sharon, 2017). Self-tracking devices are reported to be only accurate in gathering data related to particular types of activity or to particular users, while producing unreliable or even plainly wrong results for others. For example, women using wearable fitness trackers or step-counting functionalities embedded in most contemporary smartphones commonly report that some of their daily movements (e.g., pushing prams) remain unregistered or that their smartphones register different statistics when kept in handbags instead of pockets (Criado-Perez, 2020, pp.159–160; Lupton & Maslen 2018).

Technologies that collect/process data do not always account for the racial, gender and age diversity of the general population. For example, they might be more likely to produce reliable results for white, young, male users (if they were overrepresented in the development process) than for other groups (Obermeyer et al., 2019). Moreover, the functioning of algorithms and the rationale for the design of hardware employed in data-collecting devices is rarely disclosed by developers (Crawford et al., 2015). This has implications for those arguing that the genealogy of data needs to be untangled by *researchers* (Mauthner, 2019). However, such views seems to overlook the fact that untangling genealogy might not be always possible, especially when companies with commercial interests hide the exact technical specifications of their devices and algorithms, or even attempt to mislead users (and researchers) about the actual operations of their technologies by hiding relevant information in purposefully unclear terms of service and privacy policy documents (Kreitmair & Cho, 2017; Danaher et al., 2018). Therefore, it is reasonable to argue that biases inherent in devices and algorithms used for collecting and processing data make it likely for the generated big data sets to be biased as well. However, since data is framed as accurate and objective, and potential biases or limitations are not always diligently disclosed, it is difficult for researchers to identify potential biases of generated data sets.

III) Users’ and third-parties’ financial/non-financial conflicts of interests exacerbate biases. Crowdsourcing platforms such as CrowdFlower, Clickworker, Toluna, and Amazon’s Mechanical Turk are regularly used by social scientists to generate big data sets. When crowdsourcing platforms are used, financial incentives offered to participants (a payment per completed survey) and the lower cost of data collection for researchers (who incur lesser costs than when collecting data manually) might not only contribute to, but also encourage unethical practices (Quinton & Reynolds, 2017). Research subjects might decide to increase their profits by completing surveys hastily to maximize completed surveys per day or researchers might exploit subjects by not fully informing them about the required time for completing a survey, hence (inadvertently) encourage sloppy behavior and increase the likelihood of generating biased data sets (Semuels, 2018; Starkbaum & Felt 2019). Furthermore, low financial rewards offered by most crowdsourcing platforms, increases the chances of obtaining biased data sets. Crowdsourced surveys might entail non-inclusive samples as the low financial rewards do not incentivize individuals from high income countries, whereas for individuals based in low-income countries, working full time on crowdsourcing platforms could yield sufficient incomes.^9^

Moreover, when big data sets are generated using social networking sites, it might be impossible to isolate data sourced from fake and bot accounts, some of which might have been created with specific financial and political agendas. Consequently, the information contained within such data sets might have been subject to manipulation by third-parties engaged in disinformation campaigns, or otherwise tainted by trolls and malicious actors.

### Risks Arising from Reuse of Data

Social scientists commonly reuse data sets generated for other studies (Curty, 2016). In fact, Resnik’s principles of openness and efficiency demand that data sets should be made openly available and reused. However, reusing big data sets in SSR to uphold these two principles might contribute to, and even facilitate violations of other principles, as we demonstrate in this section. Although some of these issues might be connected to individual and social harms, as well as prejudices and biases discussed in the neighboring subsections, we believe that highlighting involved risks when openly available data is reused by third-parties (e.g., other researchers or non-academic parties) is essential.

Administrative data generated by public institutions is particularly useful for SSR, especially when they are in the public domain and contain demographic and financial information (Connelly et al., 2016). For instance, the European Union Open Data portal (https://data.europa.eu/euodp/en/data/) contains 1,306,410 data sets (as per February 2022) ranging from national opinion trends to medicine, mobility, demographic and gender issues.^10^ The American equivalent, the Data.gov catalog (https://catalog.data.gov/dataset), contains 341,876 data sets (as per February 2022) pertaining to various topics from property sales per county to health status for groups of Medicare beneficiaries. Besides gaining access to data that might be impossible to collect without public/governmental resources, using advanced big data analytic techniques, social scientists can extract useful information from these data sets without having to engage in time-consuming or costly data collection efforts.^11^ From an ethical perspective, this extent of availability of data sets creates three dilemmas.

I) Although reusing data sets is efficient, it has a significant (epistemic) downside: researchers have not been involved in the data collection processes, so they have no influence on, and potentially limited insight into how data was collected. Accordingly, researchers are unable to anticipate and account for undisclosed biases embedded in data sets. Especially in cases where data sets are not linked with a published manuscript or lack supplementary information about the used methodology, researchers are unaware (and unable to become aware) of biases and limitations (Mittelstadt & Floridi, 2016; Lazer et al., 2014). Hence, researchers cannot determine whether the data was collected diligently and responsibly (Wallis & Borgman, 2011), which poses a threat to the integrity of research.
II) While public availability of data enables the critical scrutiny and assessment of results and facilitates efficiency, it also makes data vulnerable to unethical practices or, worse, accessible to abusive actors. Besides benefiting academic scholars, the regulatory push for making research data FAIR (Findable, Accessible, Interoperable, Reproducible) has also allowed various non-academic parties to benefit from free research data (Wilkinson et al., 2016). When reusing data, non-academic users might not necessarily adhere to norms and values that academic researchers are expected to uphold. Researchers are (usually) required and mandated by institutions to attend research ethics and integrity trainings and have their proposals and methodology vetted by IRB or ethics committees. However, since mechanisms for regulating non-academic research are generally less rigorous (Polonetsky et al., 2015), data availability might contribute to unforeseen ethical challenges. While the number of data sets stored on repositories such as The European Union Open Data portal and the American data catalogue shows researchers’ and public institutions’ willingness to share data sets, citizens should be concerned about who will reuse these data sets and for what purposes. Furthermore, data sets are vulnerable to cyber-attacks and so-called data leaks. Even when data sets generated through research practice are seemingly protected, corrupt researchers (Cass, 1999) or other non-academic parties might steal existing data or hack data repositories to extract valuable information (Mello, 2018).
III) Data availability also facilitates data aggregation and reaching unforeseen conclusions. Whereas a study might be focused on people’s mobility patterns or earning potential, by combining/enriching results with datapoints retrieved from other data sets, possibilities to make seemingly meaningful conclusions are multiplied. For example, administrative data sets employed to determine citizens’ earnings might be linked with data about the distribution of people with particular social or ethnic background in communities, thereby allowing researchers to find correlations and arrive at prejudiced conclusions that they would not have reached if information triggering such questions would not have been readily available.^12^ Accordingly, social scientists employing big data sets generated by public institutions, shared by other researchers, or provided by commercial companies, might inadvertently violate principles of research integrity (e.g., by using data for specific objectives without subjects’ consent).^13^

These three dilemmas are further intensified because most citizens who engage in online interactions rarely understand or are informed about potential uses of their information in future research projects. Accordingly, different views are debated: While some argue that utilizing information in ways that go beyond reasonable user expectations is a violation of privacy (Nissenbaum & Patterson, 2016), others believe that research subjects should be directly prompted about data reuse (Mannheimer et al., 2019). Either way, since the notion of reasonable user expectation is open to interpretation, and, reaching out to subjects of past projects is not always possible, in practice, the onus seems to be on data collectors to anticipate and/or communicate potential reuse, or to revise their ethics protocols with amendments and obtain consent if necessary (Remenyi et al., 2011).

### Individual and Social Harms

In cases where SSR exposes participants’ personal characteristics and vulnerabilities (Nissenbaum & Patterson, 2016), using big data sets might enable researchers to predict participants’ future behavior (and behavioral patterns), which complicates upholding principles of respect for subjects and social responsibility.^14^ When predictive research efforts are coupled with commercial interests, they have resulted in unfair exclusion of vulnerable groups from opportunities (e.g., access to credit) or led to predatory marketing campaigns (Madden et al., 2017). These practices are particularly egregious when research results rationalize policies and practices to target or even discriminate against a particular group through data categorization – a viable practice even when data is anonymized (Ajana, 2017).^15^ In fact, some who argue that there is much more information available about us online than we might realize, have directly linked this issue with political power and claimed that this abundance of information makes democracies vulnerable (the more is known about each of us, the more predictable we become and hence, our political choices become more predictable) (Véliz, 2020).

Consequently, uncertainties associated with the (future) processing of data sets might impede researchers’ ability to uphold principles of social responsibility and respect for subjects. In employing big data sets, researchers or other users may employ data processing methods to achieve objectives that participants had not consented to or worse, use the data against participants’ social/political/financial interests without any regulatory oversight. Examples include zip code categorization to prioritize services (e.g., by providing faster delivery times to neighborhoods predominantly populated by wealthy white customers, cf. Ingold and Soper 2016), gerrymandering to change the political dynamic of communities, or increasing insurance premiums based on demographic segmentation of communities (Duchin, 2019).

Use of big data sets has also facilitated questionable research practices such as HARKing (Hypothesising After Results are Known), and question trolling that involves searching data with several constructs or relationships to find notable results (Kerr, 2016; Murphy & Aguinis, 2019). From a methodological perspective, these practices suggest a move from a hypothesis-driven to a hypothesis-free research paradigm (Pasquetto, 2018) – sometimes called the end of social theory (Anderson, 2008) – but they also challenge ethical principles of respect for subjects and social responsibility. While both HARKing and question trolling nullify individuals’ consent (e.g., by formulating questions/hypotheses that were not communicated to subjects in information sheets), in SSR they may also exacerbate harmful effects of research on the society through giving more control (over individuals/societies) to those who can access and/or analyze users’ data.

In terms of the principle of respect for subjects, some projects “scoop up personal information” from users’ online activities or even fitness trackers (Madden et al., 2017, p. 64). This information is then combined with personal evaluation metrics (e.g., credit history, criminal background records, educational testing scores) to tag users with specific characteristics, thereby governing users’ access or privileges (especially low-income people) in relation to various public and private services (e.g., education, insurance). These practices create digital representations of individuals as well as groups of individuals, sometimes called data doubles (Haggerty & Ericson, 2000; Ruckenstein, 2014). These data doubles are created through pattern recognition methods and then used at a massive scale to create predictive behavioral models (Fire, 2014). Subsequently, data scientists willing to engage in HARKing only need to look for patterns in data sets (also called data mining). These data mining methods are commonly used by social scientists aiming “to maximize the overall predictive power” in testing social/psychological hypotheses (Attewell et al., 2015, p. 14). The unrestricted processing of data about the behavior of large groups (or clusters within groups) might expose characteristics, vulnerabilities, and reveal the decision-making processes of specific cohorts, thereby putting them at a weaker position in comparison with researchers, institutions or companies that have access to and can interpret these results. Such knowledge about cohorts’ decision making might allow parties with financial or political agendas to target studied groups with specific strategies based on cohorts’ predicted behavioral profile, allowing them to engage, for example, in manipulation aided by information derived through HARKing.

In relation to the principle of social responsibility, the high global environmental costs of big data storage and processing are rarely considered when discussing the ethical impact of big data analytics. Crawford (2021) argues that euphemistic terms such as *cloud computing* can make us falsely believe that data-processing algorithms function in a sleek and frictionless manner. Crawford adds, devices used to store and process big data are constructed using large quantities of rare minerals, which means that their extraction leaves disastrous effects on the environment and local communities of mined areas. Additionally, these devices consume enormous amounts of electricity and exacerbate the climate crisis. Material and energy requirements are also relevant from the standpoint of the principle of efficiency as in many cases, the use of big data methods might not be the most efficient way of allocating resources when the overall environmental impact of a study is considered.

Furthermore, the distance between researchers and subjects might contribute to individual harms. Researchers involved in big data research do not directly engage with people described by the data, as opposed to SSR that involves interviews, focus groups or surveys that do not result in big data sets. For example, when studying patients’ self-reported feelings about long-term cardiac treatment, Lomborg et al., (2020) noted that as a result of interviews, researchers felt connected to subjects and their situation. Although these researchers had access to detailed information about subjects’ emotional dispositions and medical history (supplied by data collecting devices), they only recognized personal dimensions of research during direct contact with subjects.^16^ Big data SSR, however, might not necessarily require personal contact with subjects. The ethical concern being that big data’s *technological mediation* increasingly detaches researchers from participants and dilutes their perception of human subjects (Zimmer, 2018). Involved researchers might forget that specific data points within data sets are connected to subjects with expectations, rights and vulnerabilities that should be respected. Consequently, subjects are more likely to be harmed through objectification, instrumentalization of their data.

## Suggestions for Developing Ethics Guidelines

In this paper, we have argued that big data SSR involves distinct ethical issues related to prejudices and biases, risks arising from publication and reuse of data, and individual and social harms. We showed that these ethical issues complicate and/or impede researchers’ adherence to principles of honesty, carefulness, openness, efficiency, respect for subjects and social responsibility as articulated in Resnik’s research ethics framework.

Despite a wide range of potential ethical issues in big data SSR, these issues have received relatively little regulatory and ethical scrutiny. While some codes of conduct note individual ethical issues relevant to big data SSR, they rarely capture complexities of this field to a satisfactory degree and are neither globally endorsed nor enforced. Consequently, researchers willing to uphold ethical standards in conducting big data SSR might find it difficult to find relevant ethical guidance. As mentioned in Sect. 2 of this paper, in the absence of comprehensive and universally accepted research ethics procedures regarding big data SSR, research ethics committees are not subjecting big data SSR to appropriate ethical scrutiny as they currently lack the tools and knowledge necessary to do so in a satisfactory manner.

As the volume, variety and velocity of big data increases, the possibility of harnessing information from big data sets for the purposes of SSR will prove more appealing to researchers. To the best of our knowledge, this paper is the first attempt to adopt a research ethics normative framework to explore the complicated landscape of ethics of big data SSR. We believe that it should serve as a call to action for the scientific community and regulatory bodies to devote more attention to the growing complexity and variety of ethical aspects of big data SSR. The formulation of clear guidelines for big data SSR, would be one of the first steps required to reduce the likelihood of ethical issues. In line with issues identified using Resnik’s framework, we provide the following considerations to observe in developing future guidelines about big data SSR:

1. Prejudices and biases.

- When sharing their datasets as a stand-alone research output or as part of a manuscript, researchers should disclose limitations and biases of generated/reused data sets. In the absence of such information, adding disclaimers should be mandatory.
- Data repositories should mandate and prompt researchers to disclose limitations and biases when storing data sets (e.g., by adding a new mandatory textbox to fill).
- Funders, academic/non-academic research institutions and IRB/research ethics committees should provide guidance and best practices on how to minimize biases embedded in data sets and third-party technologies, and those resulting from researchers’ personal prejudices.

2. Reuse of big data and the associated risks.

- Researchers should be required to obtain research subjects’ *explicit* consent for the use of their information in big data SSR, as well as for the possibility of future reuse of their information by other studies with the possibility to opt out of future use of their data.
- Funders, academic/non-academic research institutions and IRB/research ethics committees should mandate researchers to inform their subjects about the consequences of the openness of data and instruct them about the likely future uses of data.
- Data repositories should assign a DOI for every stored data set (and their subsequent versions) to enable and encourage researchers and data watchdogs to improve dataset tracing.

3. Individual and social harms.

- Researchers should be required to follow procedures that anticipate and determine potential social and individual impacts of their study and results (e.g., by performing an anticipatory analysis similar to those gaining popularity in ethics of technology, cf. Brey, 2012).
- Funders, academic/non-academic research institutions and IRB/research ethics committees should mandate researchers to *explicitly* inform their subjects about the potential social impacts of studies employing their data.
- Researchers employing big data tools should consider local and environmental impacts, and choose providers while considering their environmental footprints, sustainability of supply chains and efficiency of adopted methodologies.

## Footnotes

1 Our working definition of big data is: Large sets of data compiled from various sources (e.g., existing administrative data, online interactions, data collected by devices) and stored in a digital form to be analyzed with computers. Big data has been characterized by three v’s: volume (the large amount of information), variety (the diverse scope of information) and velocity (the high speed at which new data is generated and analyzed) (Kitchin & McArdle, 2016).

2 While Weinhardt’s study claims to address ethical issues in big data SSR, in our view, it does not explore a single ethical issue that is unique to big data SSR. Examples he uses to illustrate social dimensions of big data research are less specific than what we describe in this paper. For example, while “the development of stock prices around the world, the tracking of trucks in automated toll systems for real-time forecasting of GDP developments, or the extraction of rental housing market information from websites and dedicated portals to estimate the development of rents over time” (Weinhardt, 2020, p. 358) could be interesting subjects for big data research, they are unrelated to the interpretative nature of SSR and only focus on hypotheses that require big data. Salganik’s book *(Bit by Bit)*, on the other hand, not only mentions but also elaborates on ethical issues of big data SSR. Salganik uses the four principles of Respect for Persons, Beneficence, Justice and Respect for Law and Public Interest introduced in The Menlo Report for *ICT research* (Dittrich & Kenneally, 2012). Since the Menlo report is built on the Belmont report, it is a better fit for computational *biomedical* research. Consequently, Salgnik too neglects ethical challenges introduced by the interpretive nature of SSR and the potential for prejudices and biases. In addition to highlighting ethical issues linked to the interpretative nature of SSR, our work specifically discusses ethical issues related to research integrity and environmental sustainability.

3 Two peer-reviewers brough these special issues to our attention.

4 As discussed by González-Bailón (2013), tools focusing merely on the content of the processed information can neither account for the context, nor consider the agency of people involved.

5 We use the term *symbolic violence* after Bourdieu to designate non-physical harms, such as derogatory or stigmatizing language, social exclusion, and lack of representation, which are inflicted upon individuals with the purpose of entrenching the existing stratification of society and the associated inequality and injustice (Bourdieu, 1991; Bourdieu & Wacquant, 1992).

6 The European Commission has published specific guidelines entitled *Ethics in Social Sciences and Humanities* (European Commission, 2018). This document highlights ethical issues relevant to data collection efforts that are internet-mediated and/or use social media, but it does not capture all issues raised in this article. Furthermore, endorsed by the academy of sciences in more than 40 countries, the European Code of Conduct for Research Integrity specifically notes that “researchers, research institutions and organizations provide transparency about how to access or make use of their data and research materials” (ALLEA, 2017, p.6). While the notion of ‘how to make use of their data and research materials’ could also imply disclosure of biases and limitations of data sets to facilitate ethical use of data, to the best of our knowledge, none of the major repositories (even the EU Open Data portal) require such disclosures. Although these kinds of disclosures are more common in published manuscripts (wherein study limitations are mentioned), similar practices have not been suggested for data sets.

7 Self-tracking technologies include devices and smartphone apps that enable users to collect data about themselves and their daily activities (Neff & Nafus, 2016). Popular examples include Fitbit fitness bands and Apple Watch that collect information about users’ physical activity, sleep patterns and mood. Since these technologies enable the collection of a variety of behavioral information about subjects with little difficulty and costs, they benefit SSR. For example, Lomborg et al., (2020) used Fitbits to study how live monitoring of heartrate could impact cardiac patients’ mood, while also discussing patients’ skills and cultural contexts when making sense of their medical information.

8 As self-tracking technologies reduce qualitative phenomena to their quantifiable characteristics, they often fail to provide contextual factors that could be relevant for the assessment of the information in generated data set.

9 Crowdsourcing platforms can be seen as inherently exploitative. For example, Crawford (2021) observed that many users of crowdsourcing platforms receive less than their local minimum wage for their contributions. Since platforms like Mechanical Turk can be the main source of income for some people, and as these platforms often effectively outsource data collection to regions where labor is much cheaper, researchers should envisage that lowering the *financial cost* of conducting research might have high *ethical costs*.

10 A regulatory push from the European Commission to “make as much information available for re-use as possible” by public agencies/institutions has increased availability of data sets (European Commission 2020, paragraph 1). Additionally, due to the international support and mandates for Open Access publication of data to realize the ambition of “open research data per default (but allowing for opt-outs)” (European Commission n.d., paragraph 3), results and the *data associated* of thousands of research projects are publicly available for reuse.

11 In the US, this trend was exacerbated when in 2013 the Obama Administration made open data the default method of disseminating research conducted by the federal government. Accordingly, data sets that include information on health, climate, small business and manufacturing opportunities, crime, education, and public domain information on the federal workforce should be made publicly available. Marion Royal (the director of data.gov) notes that “the model of preserving privacy by individual consent might be obsolete when so much data is passively captured by sensors, and the abundance of social media and search data collected by private companies makes anonymization ‘virtually impossible,’ … Privacy as a concept is becoming less clear as technology increases and big data becomes more prevalent, and available” (Mazmanian, 2014, paragraph 4–7).

12 As internet companies commonly track cookies across multiple websites to collect users’ data (e.g., Facebook has admitted to collecting data even on non-members by tracking cookies across partnering websites, cf. Brandom, 2018), it is often practically impossible for users to establish which data was willingly and knowingly shared. Moreover, since data is exchanged among a wide range of vendors, it is virtually impossible to determine a full life cycle or value chain of users’ data. For instance, The New York Times website lists among its “nonessential” cookies 19 marketing and 8 advertising trackers which send information about readers’ activity to companies such as Google, Facebook, Microsoft (cf. https://www.nytimes.com/privacy/cookie-policy) with access to enormous datasets and capability to process/aggregate data. Innocuous data about reading habits could be used to target specific groups with e.g., marketing/political campaigns across other platforms.

13 In 2013, the New York City Taxi & Limousine Commission released an anonymized dataset with information about 173 million individual cab rides – including pickup and drop-off times, locations, fare and tip amount. After the release, researchers that freely accessed the database were able to reveal private and sensitive information about the taxi-drivers (e.g., religious belief, average income and even an estimation of their home address), thus demonstrating the ease with which databases can be processed to reveal information about individuals (Franceschi-Bicchierai, 2015).

14 Practices such as psychographic targeting that involve targeting users based on their personality traits (Gibney, 2018), or the Big Five scale test that measure users’ five personality traits (i.e., openness, conscientiousness, extraversion, agreeableness and neuroticism) based on their Facebook likes (Kosinski, 2013), are among methods that allow predicting but also influencing human behavior.

15 In data categorization practices, individuals are targeted not based on unique characteristics (e.g., browsing data or employment history) or identifying features (e.g., biometric data), but as a result of their membership of a group purported statistically more likely to exhibit certain behaviors. For example, financial institutions could (unfairly) deny a loan to an individual because according to their data, people belonging to the individual’s ethnic or social group are statistically more likely to default on loans.

16 Interestingly, Lomborg et al., (2020) also noted that they were not required to obtain ethical approval for their research despite being intimately involved in their subjects’ lives.
